# Supplementary material for: Causal structure of interacting Weyl fermions in condensed matter systems
Source: Nat Commun. 2023 Apr 19;14:2228. doi: 10.1038/s41467-023-37931-w (PMC10115776; doi:10.1038/s41467-023-37931-w)
Supplement: Supplementary file 1 — Supplementary Information [file 41467_2023_37931_MOESM1_ESM.pdf]

Supplementary Information: Causal structure of interacting Weyl fermions in condensed matter systems

Wei-Chi Chiu *,[1] Guoqing Chang *,†,[2] Gennevieve Macam,[3,4] Ilya Belopolski,[5,6] Shin-Ming Huang,[4] Robert Markiewicz,[1] Jia-Xin Yin,[7] Zi-Jia Cheng,[5] Chi-Cheng Lee,[8] Tay-Rong Chang,[9,10,11] Feng-Chuan Chuang,[4,11,12] Su-Yang Xu,[13] Hsin Lin,[14] M. Zahid Hasan,[5,15] and Arun Bansil[1]

[1]*Department of Physics, Northeastern University, Boston, MA 02115, USA*

[2]*Division of Physics and Applied Physics, School of Physical and Mathematical Sciences, Nanyang Technological University, 21 Nanyang Link 637371, Singapore*

[3]*National Institute of Physics, University of the Philippines, Diliman, Quezon City 1101, Philippines*

[4]*Department of Physics, National Sun Yat-sen University, Kaohsiung 80424, Taiwan*

[5]*Laboratory for Topological Quantum Matter and Advanced Spectroscopy (B7), Department of Physics, Princeton University, Princeton, New Jersey 08544, USA*

[6]*RIKEN Center for Emergent Matter Science (CEMS), Wako, Saitama 351-0198, Japan*

[7]*Department of Physics, Southern University of Science and Technology, Shenzhen, Guangdong 518055, China*

[8]*Department of Physics, Tamkang University, Tamsui, New Taipei 251301, Taiwan*

[9]*Department of Physics, National Cheng Kung University, Tainan, Taiwan*

[10]*Center for Quantum Frontiers of Research and Technology (QFort), Tainan, Taiwan*

[11]*Physics Division, National Center for Theoretical Sciences, Taipei 10617, Taiwan*

[12]*Department of Physics, National Tsing Hua University, Hsinchu 30013, Taiwan*

[13]*Department of Chemistry and Chemical Biology, Harvard University, Cambridge, MA, USA.*

[14]*Institute of Physics, Academia Sinica, Taipei 115201, Taiwan*

[15]*Lawrence Berkeley National Laboratory, Berkeley, California 94720, USA*

---

*These authors contributed equally to this work.

†Corresponding author (email): guoqing.chang@ntu.edu.sg

This PDF file includes:

Supplementary Discussions:

Weyl-CDW interacting when the nodes lie at the same energy

The effect of CDW strength

Causal structure for Weyl nodes with non-linear dispersion

Causal structure in $\mathbf{k}\cdot\mathbf{p}$ description

Supplementary Figs. 1 to 6

WEYL-CDW INTERACTING WHEN THE NODES LIE AT THE SAME ENERGY

We consider the case $A = 0$, where four Weyl nodes at $\pm(\pi/2, 0, \pm\pi/2)$ are at the same energy ($E_{\mathrm{W}_1} = E_{\mathrm{W}_2}$) (Supplementary Fig. 1a). After including the Peierls' dimerization with the CDW strength $\delta = 0.05$, each pair of Weyl nodes along the k_x-direction are folded to the same point ($\mathbf{Q}_{\mathrm{CDW}} = \mathbf{k}_{\mathrm{W}_1} - \mathbf{k}_{\mathrm{W}_2} = (\pi, 0, 0)$). As a result, the Weyl nodes annihilate with each other and open a global gap between the conduction and valence bands in the reduced BZ (Supplementary Fig. 1b). Now, we consider another interacting scenario where CDW Q-vector is different from the separation of Weyl nodes ($\mathbf{Q}_{\mathrm{CDW}} \neq \mathbf{k}_{\mathrm{W}_1} - \mathbf{k}_{\mathrm{W}_2}$) (Supplementary Fig. 1c). By choosing $k_1 = 1.1\pi/2$, the separation vector for the pair of Weyl nodes along the k_x-direction is $(1.1\pi, 0, 0)$. After the inclusion of a CDW interaction with $\delta = 0.05$, the paired Weyl nodes are folded at different locations in momentum space. Weyl nodes do not annihilate with each other and the system remains in the semimetal phase (Supplementary Fig. 1d).

THE EFFECT OF CDW STRENGTH

Here, we demonstrate how the CDW interaction δ attracts the two Weyl quasiparticles toward each other and may make the system cross the quantum critical point. We first use the same parameters, $A = 0.3$, $\theta = 0$, $k_1 = 1.3\pi/2$, for the momentum-like case in Fig. 1e but change the CDW strength δ (Supplementary Fig. 2a). We can see that the CDW interaction tends to push two Weyl nodes toward each other when increasing δ increase (Supplementary Fig. 2b). When the $\delta = 0.17$, the system reaches the quantum critical point, but the whole system remains gapless (Supplementary Fig. 2c). Once the CDW

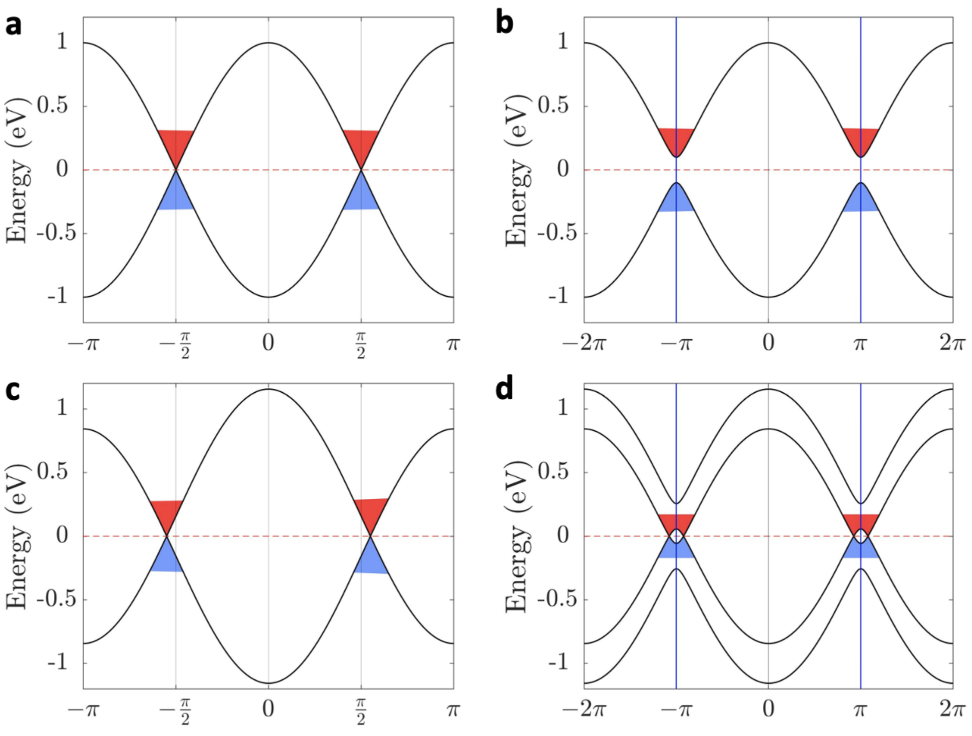

Supplementary Fig. 1. **Weyl-CDW interacting when the nodes lie at the same energy.** **(a-b)** Band structure as a function of k_x. $A = 0$, $\theta = 0$, $k_1 = \pi/2$, $k_y = 0$, and $k_z = \pi/2$. **a**. Without CDW, four Weyl nodes are at the $\pm(\pi/2, 0, \pm\pi/2)$ with no energy difference. The red/blue color blocks represent the conduction/valence bands of Weyl cones. **b** With CDW, CDW Q-vector is along $(\pi, 0, 0)$ and the CDW strength $\delta = 0.05$. Weyl nodes with opposite chirality are nested exactly to the same point in energy-momentum space. There is a global gap opened by CDW between conduction and valence bands. The blue lines represent the boundary of the reduced BZ. **(c-d)**. Bands structure along the k_x with $A = 0$, $k_1 = 1.1\pi/2$, $k_y = 0$, and $k_z = \pi/2$. **c** Without CDW, four Weyl nodes are at the $\pm(1.1\pi/2, 0, \pm\pi/2)$ **d**. With CDW, CDW Q-vector is along $(\pi, 0, 0)$ and the CDW strength $\delta = 0.05$. The Weyl nodes cannot be gapped and the system remains in the semimetal phase.

strength δ is larger than 0.17, the system is driven from momentum-like to energy-like, and it undergoes a topological phase transition (Supplementary Fig. 2d). Note that we consider a case with a larger separation in energy-momentum space between two interacting Weyl quasiparticles, so it requires a relatively large CDW strength $\delta = 0.17$, compared to the hopping strength 0.5 (see the $\frac{1}{2} \pm \delta$ terms in Eq. (12) of the Methods section), to reach the quantum critical point. However, when the two Weyl nodes are very close to each other's

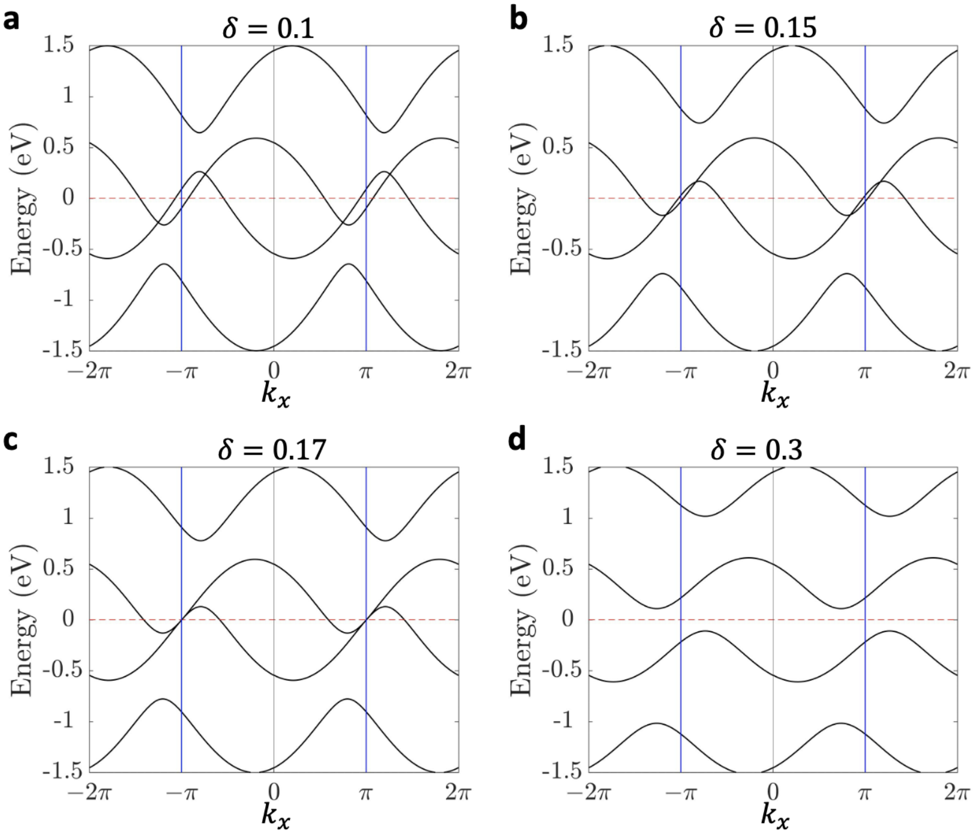

Supplementary Fig. 2. **CDW-driven momentum-like to energy-like crossover**. Bands structure as a function of k_x with $A = 0.3$, $\theta = 0$, $k_1 = 1.3\pi/2$, $k_y = 0$, and $k_z = \pi/2$. The blue lines represent the boundary of the reduced BZ. CDW Q-vector is along $(\pi, 0, 0)$. **a** CDW strength $\delta = 0.1$. The system is momentum-like and remains in the Weyl semimetal phase. **b** CDW strength $\delta = 0.15$. The larger CDW strength drives two Weyl nodes closer to each other. **c** CDW strength $\delta = 0.17$. It's the quantum critical point of the topological phase transition. **d** CDW strength $\delta = 0.3$. When $\delta > 0.17$, the CDW strength is large enough to drive the whole system from momentum-like to energy-like and the system, therefore, undergoes a topological phase transition.

dispersion cone (but still in the momentum-like region), even a small CDW strength (one order of magnitude smaller than the hopping strength) can make the two Weyl quasiparticles fall into each others' dispersion cone and become energy-like (and the system is gapped). It's worth emphasizing that our argument, that a topological phase transition can occur only when a pair of interacting Weyl nodes with opposite topological charges are energy-like, remains correct.

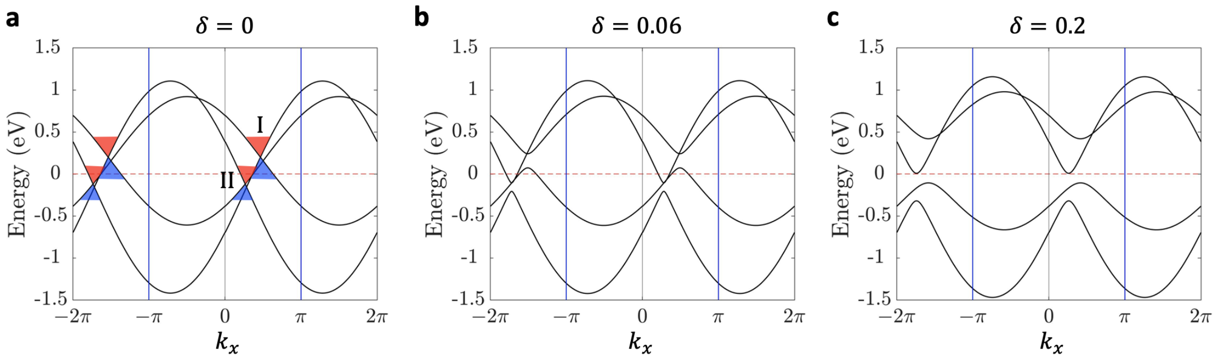

Supplementary Fig. 3. **The effect of CDW strength in quantum horizon region**. Bands structure as a function of k_x with $A = 0.3$, $\theta = 1$, $k_1 = 1.1\pi/2$, $k_y = 0$, and $k_z = \pi/2$. The blue lines represent the boundary of the reduced BZ. **a** Without CDW ($\delta = 0$), it's the case of quantum horizon region as shown in Fig. 4b in the main text. **b** With CDW, CDW Q-vector is along $(\pi, 0, 0)$ and the CDW strength $\delta = 0.06$. The Weyl nodes remain and there is no metal-insulator phase transition. It's the case when Weyl nodes II touch the dispersion cone of Weyl node I. **c** With CDW, CDW Q-vector is along $(\pi, 0, 0)$ and the CDW strength $\delta = 0.2$. A global gap opened by the CDW around the Fermi energy. The strong enough CDW strength drives the system from the quantum horizon region to the energy-like region.

Furthermore, we show the effect of tuning CDW strength in the quantum horizon region. We use the same parameters, $A = 0.3$, $\theta = 1$, $k_1 = 1.1\pi/2$, for the case in Fig. 4b in the main text but change the CDW strength δ (Supplementary Fig. 3a). When the $\delta = 0.06$, the Weyl nodes II touch the dispersion cone of Weyl node I but the whole system remains in the quantum horizon region (Supplementary Fig. 3b). When the CDW strength is larger than 0.06, the system will be driven to the energy-like region and open a global band gap (Supplementary Fig. 3c). Note that, in this case, the CDW strength required to drive the system to fall into the energy-like region is one order smaller than the hopping strength, so it is close to the quantum critical point as we mentioned in the previous paragraph.

CAUSAL STRUCTURE FOR WEYL NODES WITH NON-LINEAR DISPERSION

Here, we consider the case where the non-linear dispersion of the Weyl cones is not negligible (Supplementary Fig. 4a), and the two interacting Weyl quasiparticles locate in

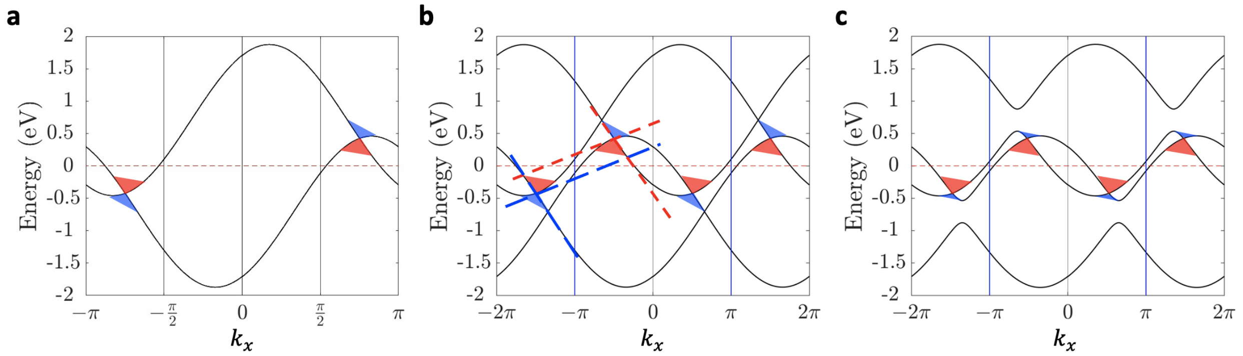

Supplementary Fig. 4. **The non-linear dispersion of Weyl nodes**. **(a-c)**. Bands structure as a function of k_x with $A = 0.6$, $\theta = 0$, $k_1 = 1.5\pi/2$, $k_y = 0$, and $k_z = \pi/2$. **a** Without CDW, the dispersion cones of the two Weyl quasiparticles become non-linear. **b** Without CDW ($\delta = 0$), the folded bands in the double supercell BZ along x-direction. The red (blue) dashed line shows the linear approximation of the dispersion cones of the Weyl node at higher (lower) energy. **c** With CDW of Q-vector along $(\pi, 0, 0)$ and the CDW strength $\delta = 0.05$: The Weyl nodes remain intact and there is no global band gap between valence and conduction bands.

the region where each others' dispersion cone becomes non-linear as shown in Supplementary Fig. 4b. In this case, the gapping condition of Eq. (3) in the main text based on the linear approximation of Weyl cones indicates that the two interacting Weyl nodes are energy-like (the blue and red dashed lines in Supplementary Fig. 4b). However, the two interacting Weyl nodes lie out of each other's dispersion cone. When a non-zero CDW interaction is included ($\delta = 0.05$), there is no global band gap between the conduction and valence bands (Supplementary Fig. 4c). This result indicates that the Eq. (3) in the main text can be further generalized to the case simply based on whether two interacting Weyl quasiparticles are inside or outside of each other's dispersion cone, even if it is non-linear, as follows:

$$\begin{cases} \text{Both outside}: \text{ Momentum-like} \\ \text{Both inside}: \text{ Energy-like} \\ \text{One inside, one outside}: \text{ quantum horizon region} \end{cases} . \tag{1}$$

CAUSAL STRUCTURE IN $\mathbf{k}\cdot\mathbf{p}$ DESCRIPTION

Besides considering a specific model and having the classification of the causal structure (phase diagram) of the interacting Weyl quasiparticles system, here, we discuss each case from the low-energy effective $\mathbf{k}\cdot\mathbf{p}$ Hamiltonian near the two interacting Weyl nodes. When two Weyl nodes with opposite chirality lie close to each other in energy-momentum space, the linear expansion around each Weyl node can be expressed in the following form

$$H_1(\mathbf{k}) = E_1\sigma_0 + v_1\boldsymbol{\sigma}\cdot(\mathbf{k}-\mathbf{p}_1), \tag{2}$$

$$H_2(\mathbf{k}) = E_2\sigma_0 - v_2\boldsymbol{\sigma}\cdot(\mathbf{k}-\mathbf{p}_2). \tag{3}$$

Here, $\boldsymbol{\sigma}$ are the Pauli matrices, v_1 and v_2 are two positive values for the Fermi velocity of the Weyl quasiparticles and E_1, E_2, $\mathbf{p}_1$ and $\mathbf{p}_2$ determine the locations of Weyl quasiparticles in energy-momentum space. The sign in front of the v_1 (v_2) explicitly codes the sign of the topological charge associated with the Weyl node. Thus, the simplest Hamiltonian that describes these two interacting Weyl nodes is:

$$H = \begin{bmatrix} H_1(\mathbf{k}) & m \\ m & H_2(\mathbf{k}) \end{bmatrix}. \tag{4}$$

where the off-diagonal entry m couples the two Weyl nodes with opposite chirality as an effective mass term.

We first discuss the causal structure for the interacting Hamiltonian when $m = 0$. For the energy-like case, we use the parameters: $E_1 = -0.3$, $E_2 = 0.3$, $\mathbf{p}_1 = (-0.1, 0, 0)$, $\mathbf{p}_2 = (0.1, 0, 0)$ and $v_1 = v_2 = 1$ (Supplementary Fig. 5a). When a non-zero interaction term $m = 0.2$ is included, a global gap is opened around the Fermi level between the two Weyl nodes as seen in Supplementary Fig. 5d. Note that, in the energy-like case, the global gap will open no matter how small the interaction m is. The momentum-like case is considered in Supplementary Fig. 5b with $E_1 = -0.3$, $E_2 = 0.3$, $\mathbf{p}_1 = (-0.5, 0, 0)$, $\mathbf{p}_2 = (0.5, 0, 0)$ and $v_1 = v_2 = 1$. Turning on $m = 0.2$ (Supplementary Fig. 5e) now does not open a global gap between the two Weyl nodes and the system remains in the metallic state. The case where the two Weyl nodes involve different Fermi velocities is considered in Supplementary Fig. 5c with $E_1 = -0.3$, $E_2 = 0.3$, $\mathbf{p}_1 = (-0.25, 0, 0)$, $\mathbf{p}_2 = (0.25, 0, 0)$ and $v_1 = 1$ and $v_2 = 2.5$. We can see that now a global gap does not necessarily open between the two Weyl nodes when interactions are included (Supplementary Fig. 5f). However, in this case, with a larger

interaction strength m, the two Weyl quasiparticles could enter into each others' energy-like region, so whether or not a global band gap opens up depends on the strength of m.

We further discuss the case when the non-linear dispersion is non-negligible in the local expansion region of two interacting Weyl nodes. Here, we approximate the expansion around each Weyl node up to the quadratic terms as follows:

$$H_1(\mathbf{k}) = E_1\sigma_0 + v_1\boldsymbol{\sigma}\cdot(\mathbf{k}-\mathbf{p}_1) + \lambda_1\sigma_0\left(\mathbf{k}-\mathbf{p}_1\right)^2, \tag{5}$$

$$H_2(\mathbf{k}) = E_2\sigma_0 - v_2\boldsymbol{\sigma}\cdot(\mathbf{k}-\mathbf{p}_2) + \lambda_2\sigma_0\left(\mathbf{k}-\mathbf{p}_1\right)^2. \tag{6}$$

The λ_1 (λ_2) is the coefficient corresponding to the kinetic energy term. With using Eq. (5)-(6) in Eq. (4) , we discuss the causal structure for the interacting Hamiltonian. For the energy-like case, we use the parameters: $E_1 = -0.3$, $E_2 = 0.3$, $\mathbf{p}_1 = (-0.1, 0, 0)$ and $\mathbf{p}_2 = (0.1, 0, 0)$, $v_1 = v_2 = 1$, and $\lambda_1 = \lambda_2 = 0.5$ (Supplementary Fig. 6a). When a non-zero interaction term $m = 0.2$ is included, a global gap is opened around the Fermi level between the two Weyl nodes as seen in Supplementary Fig. 6d. The momentum-like case is considered in Supplementary Fig. 6b with $E_1 = -0.2$, $E_2 = 0.2$, $\mathbf{p}_1 = (-0.5, 0, 0)$, $\mathbf{p}_2 = (0.5, 0, 0)$ and $v_1 = v_2 = 1$, and $\lambda_1 = \lambda_2 = 0.5$. Turning on $m = 0.2$ (Supplementary Fig. 6e) does not open a global gap between the two Weyl nodes and the system remains in the metallic state. The quantum horizon region case is considered in Supplementary Fig. 6c with $E_1 = -0.3$, $E_2 = 0.3$, $\mathbf{p}_1 = (-0.3, 0, 0)$, $\mathbf{p}_2 = (0.3, 0, 0)$ and $v_1 = v_2 = 1$, and $\lambda_1 = \lambda_2 = 0.5$. Note that it's not necessary to consider different Fermi velocities for the two Weyl nodes to make the two Weyl nodes in the quantum horizon region due to the non-linear dispersion. When interactions are included, the global band gap does not open between the two Weyl nodes ($m = 0.05$) (Supplementary Fig. 6f), and it does open when $m = 0.2$.

From the above discussion based on the low-energy effective Hamiltonian, we obtain the same overall causal structure in all cases, and the same criteria for gap-opening that is driven by the causal structure in energy-momentum space. Our conclusions concerning gap-opening are thus universal and independent of the choice of the model and the form of the interaction.

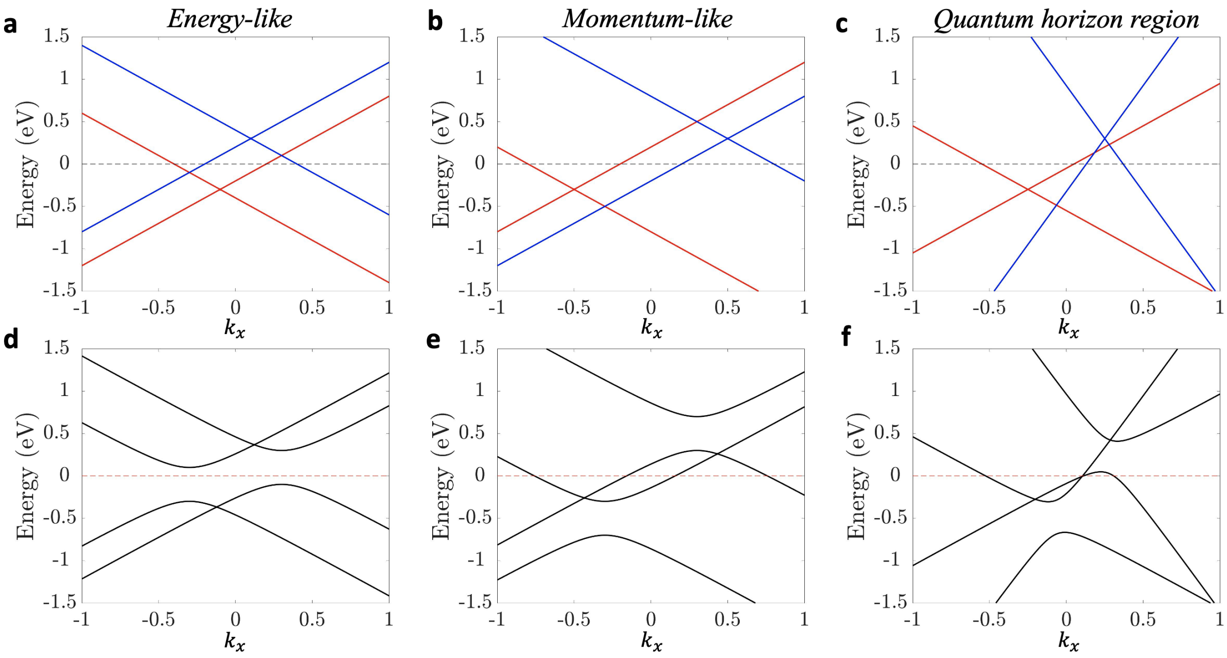

Supplementary Fig. 5. **Causal structure in k·p description**. **(a-c)** Red (blue) lines represent the linear dispersion along k_x of Weyl quasiparticles with positive (negative) topological charge located at $E_1 = -0.3$ ($E_2 = 0.3$). **a** Energy-like case with $\mathbf{p}_1 = (-0.1, 0, 0)$, $\mathbf{p}_2 = (0.1, 0, 0)$, $v_1 = v_2 = 1$, and $m = 0$. **b** Momentum-like case with $\mathbf{p}_1 = (-0.5, 0, 0)$, $\mathbf{p}_2 = (0.5, 0, 0)$, $v_1 = v_2 = 1$, and $m = 0$. **c** Quantum horizon region case with $\mathbf{p}_1 = (-0.25, 0, 0)$, $\mathbf{p}_2 = (0.25, 0, 0)$, $v_1 = 1$, $v_2 = 2.5$, and $m = 0$. **d** Energy-like case with turning on of the interaction term $m = 0.2$. **e** Momentum-like case with turning on of the interaction term $m = 0.2$. **f** Quantum horizon region case with including $m = 0.2$.

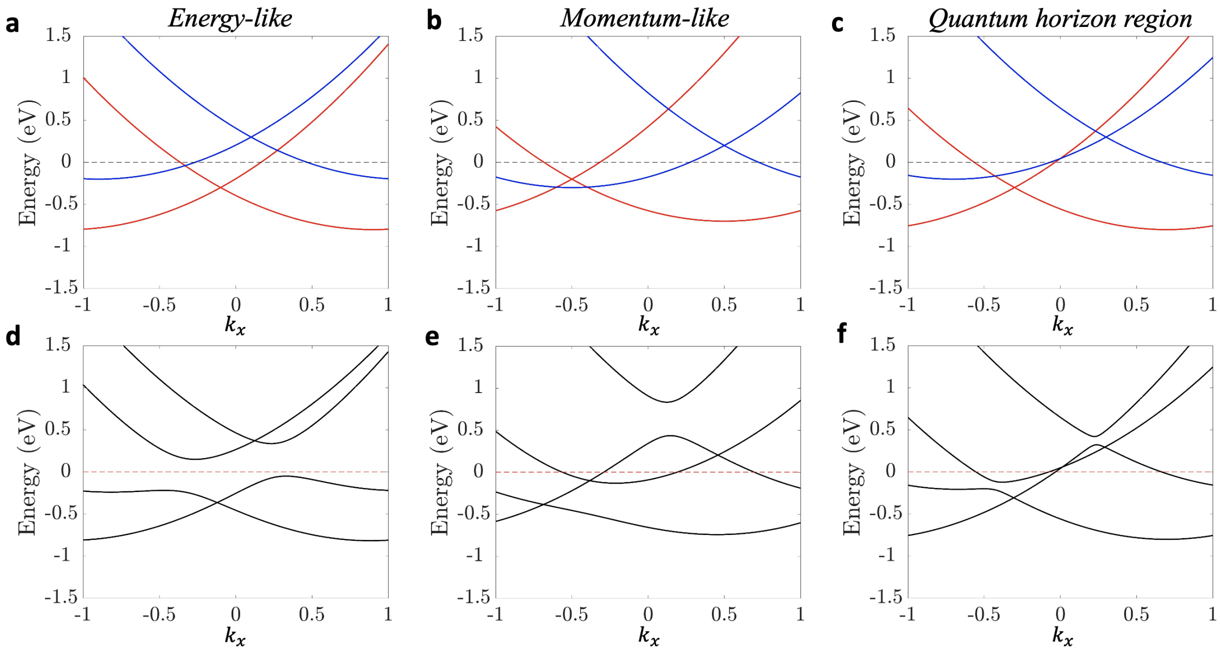

Supplementary Fig. 6. **Causal structure in $\mathbf{k}\cdot\mathbf{p}$ description with non-linear dispersion**. **(a-c)** Red (blue) lines represent the linear dispersion along k_x. **a** Energy-like case with $E_1 = -0.3$, $E_2 = 0.3$, $\mathbf{p}_1 = (-0.1, 0, 0)$, $\mathbf{p}_2 = (0.1, 0, 0)$, $v_1 = v_2 = 1$, $\lambda_1 = \lambda_2 = 0.5$, and $m = 0$. **b** Momentum-like case with $E_1 = -0.2$, $E_2 = 0.2$, $\mathbf{p}_1 = (-0.5, 0, 0)$, $\mathbf{p}_2 = (0.5, 0, 0)$, $v_1 = v_2 = 1$, $\lambda_1 = \lambda_2 = 0.5$, and $m = 0$. **c** Quantum horizon region case with $E_1 = -0.3$, $E_2 = 0.3$, $\mathbf{p}_1 = (-0.3, 0, 0)$, $\mathbf{p}_2 = (0.3, 0, 0)$, $v_1 = v_2 = 1$, $\lambda_1 = \lambda_2 = 0.5$, and $m = 0$. **d** Energy-like case with turning on of the interaction term $m = 0.2$. **e** Momentum-like case with turning on of the interaction term $m = 0.2$. **f** Quantum horizon region case with including $m = 0.05$.
